# Measuring Method for Lightning Channel Temperature

**DOI:** 10.1038/srep33906

**Published:** 2016-09-26

**Authors:** X. Li, J. Zhang, L. Chen, Q. Xue, R. Zhu

**Affiliations:** 1 Collaborative Innovation Center on Forecast and Evaluation of Meteorological Disasters, Nanjing University of Information Science & Technology, Nanjing, 210044, China

## Abstract

In this paper, we demonstrate the temperature of lightning channel utilizing the theory of lightning spectra and the model of local thermodynamic equilibrium (LTE). The impulse current generator platform (ICGS) was used to simulate the lightning discharge channel, and the spectral energy of infrared spectroscopy (930 nm) and the visible spectroscopy (648.2 nm) of the simulated lightning has been calculated. Results indicate that the peaks of luminous intensity of both infrared and visible spectra increase with the lightning current intensity in range of 5–50 kA. Based on the results, the temperature of the lightning channel is derived to be 6140.8–10424 K. Moreover, the temperature of the channel is approximately exponential to the lightning current intensity, which shows good agreement with that of the natural lightning cases.

The inner physic mechanism of the lightning channel is very important for lightning researches. It is related to the formation and development of the lightning channel^1^,^2^, and the parameter testing of lightning channel is a basic issue for detection^3^,^4^, warning and protective^5^,^6^, etc. Temperature is one of the most important channel parameter, as it is the main cause of lightning fire, electrical system damage. However, the temperature of the lightning channel is hard to be measured directly. The instantaneous peak value of energy is rather high in the lightning process^7^,^8^, about 55 kW·h energy released within several microseconds. There are massive plasmas assembling in the lightning channel whose peak temperatures reach over 10000 K, which is caused by the transient lightning discharge. The random and transient features of the natural lighting make it hard to get the temperature of the lighting discharge channel. Spectral diagnostics of the channel plasma is an effective way to obtain the temperature of discharge channel, and much research has been involved^9^,^10^.

In the previous researches, the lightning spectra were just recognized by the conventional spectrograph, furthermore, used to analyze the plasmas in the lightning channel. As the shortage of the parameters of the atomic energy level and transition, the recognition was equivocal^11^. Then the slitless spectrograph has been used to observe the spectrum of single return stroke lightning generally and it tends to be mature that the analysis of the lightning channel characteristics and the physical process which was reflected by the lightning spectra^12^. Wang Jie calculated the temperatures in diverse positions of the channel using spectrums of intracloud lightning channel in Naqu, Xizang Province^13^. Weidman *et al*. analyzed the spectral radiant characteristics of 850 nm–1400 nm spectrums of the triggered lightning, based on the wavelengths of 868.0 nm and 1011.3 nm of the neutral atoms NI spectra, reckoning that the channel temperature yields about 16000 K^14^. Qu Haiyan discussed the near infrared spectroscopy and the evolutionary characteristics of lightning channel where the temperature varies with the position of the lightning channel during the discharge process, which is based on the analysis of the spectral characteristics of the lightning near infrared spectroscopy ranging from 760 nm to 970 nm, and is measured in Shandong Province^15^. However, because of the randomness of lightning occurrence, it is difficult to receive the natural spectrums with various currents, which puzzles the lightning physical researchers^16^. In the paper we used the impulse current generator platform (ICGS) to simulate lightning channel with desired current strength and calculate the channel temperature by testing the spectrums in both infrared and visible regimes. The results have good agreement with that provided by previous researches.

## Theoretical analysis

The atomic ionization exists in gas when the absolute temperature is not 0 K, which means that there exist other charged particles besides the neutral particles. Only when the density of these particles is massive enough to form the space charge, limiting movement itself, could other charged particles affect the gas characteristics heavily.

This limitation tends to be important with the enhancement of the density. As the ample density, the macroscopic electrical neutrality is kept by the interaction between the positive and negative charged particles in a certain volume of gas (the gas volume has analogy with the space of the charged particles) whose destructive effect could induce electric field, thus the resumptive time would be shorten. Aerial discharge is one of the ways transforming gas into plasma, however not all the discharged gas could reveal the plasma characteristics. Only when the ionization is strong enough, would the plasma properties be presented. Up to dozens to several hundred kA currents could be induced by lightning instantly and such large currents could heat the lightning channel to tens of thousands K. At normal temperature, various molecules and atoms in the atmosphere would be dissociated and ionized instantaneously in such high circumstance temperature, which leads to diverse elements atoms and various levels ionized ions in the channel. Meanwhile, a mass of electrons are generated simultaneously and the whole channel is in a plasma state. Because of the complexity and the transient of the lightning discharge, it is difficult to test the physical parameters of the channel directly. The research could be implemented by the quantitative analyses of the lightning spectrum to obtain the inner physical parameters. In the analytical process, the theoretical assumption should be met as follows

(1) lightning channel is optically thin for the researched spectral lines; (2) lightning channel is in local ther-modynamic equilibrium (LTE) status.

### The LTE Model

Plasmas only satisfy the LTE to guarantee that the velocities of the plasmas meet the Maxwell distribution, each charged ion and atom conform to the Saha distribution and each energy level conform to the Boltzmann statistical distribution. Thereby the quantitative relationships having clear physical meanings are established between the radiation quantity of the plasmas and diverse state parameter^17^. The LTE condition would be tenable, only when the collision processes of electron-atom and electron-ion are achieved within several microseconds and play a leading role in the plasma velocity equation. So only when the Ne electron density is large enough, could the LTE be achieved in the system, meaning that the plasmas should meet the following LTE necessary condition


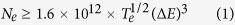


where:

*N*_e_ is the electron density of the lightning channel plasmas, eV/cm^3^; Δ*E* is the energy difference between the involved energy states; *T*_*e*_ is the temperature of electrons, K. It is often set as the measure standard that the difference between the first excited state and the ground state to ensure the energy levels meet the above conditions.

The lower excited state of NII dominates the lightning channel plasmas, the temperature range being in 2 × 10^4^ K~3 × 10^4^ K, ΔE being in 10 eV~30 eV. Based on equation (1), the plasmas satisfy the LTE condition in the channel when the electron density range from 10^17^ to 10^18^ cm^−3^. The value of the plasma density is about 10^17^ cm^−3^, which is calculated utilzing the Stark widening method. Thereout, it could be judged that the plasmas in the lightning channel satisfy the LTE condition.

According to the corresponding characteristics of the lightning spectrum, it is required to ensure the electron density in the return stroke channel that the lightning channel is optically thin. Only when the channel keep in balance on radiation and absorption could the return stroke channel be formed. Orville *et al*. analyzed the lightning spectrum by time accumulation and resolution, gaining that the ion channel was optically thin for NII particles preliminarily^17^.

### The measuring theory for lightning channel temperature

Based on the atom spectrum theory, the charged particles in lower energy state would be excited to higher energy state. Then the excited charged particles are quite unstable and would return to the lower energy state from the higher after being excited 10^−8^ s. When the excited charged particles transit from the higher energy level to the lower energy level, the energy radiated in form of light is given as


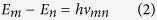


where: *E*_m_ is energy of the particles in the higher energy level; *E*_n_ is energy of the particles in the lower energy level; *m, n* are the energy levels; h is the Planckconstant; *v*_*mn*_ is the frequency of the radiation spectrum generated by the particle transition.

In the LTE condition, it is assumed that the neutral atom in the channel is in a state of excitation when the lightning channel is at a certain temperature. Based on the Boltzmannformula, the number of particles in each excited state is


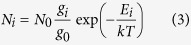


where: *N*_*i*_ is the number of particles in the excited state *i* in a unit volume of in the lightning channel; *N*_*0*_ is the number of particles in the ground state; *g*_*i*_ and *g*_0_ are the statistical weights of the excited state and the ground state; *E*_*i*_ is the excited potential, standing for the energy level of the excited state *i*, eV; *k* is the Boltzmann constant; *T* is the temperature of the lightning channel, K. From the equation (3), when the excitation temperature of the plasmas in the lightning channel is high enough, the neutral atom is easy to be excited to the higher energy level and more atoms are in the excited state. Meanwhile, the excited plasmas is unstable in the channel, and is easy to return to the ground state quickly, thus radiate photons. It is assumed that one particle should be excited to the *i* energy level, and when it recovers from the higher energy level to the lower energy level, the transitions between various energy levels have a variety of likelihood. Set the transition probability between *i* and *m* energy level as *A*_*im*_, then the difference between *i* and *m* is


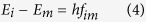


where *f*_*im*_ are the frequencies of the transition spectral lines between *i* and *m* energy level.

The transition spectral line intensities are *I*_*im*_:


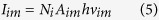


where: *I*_*im*_ are the spectral line intensities, transiting between *i* and *m* energy level, J/(cm^3^·s); *A*_*im*_ is the transition probability between two energy levels.

According to the above equations, the spectral line intensities yield to be


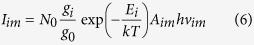


The intensity ratio of the two bands of the same kind of particle should satisfies


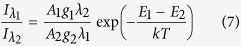


where: *I*_*λ*_1__ and *I*_*λ*_2__ are the intensities of spectral lines whose wave lengths are *λ*_1_ and *λ*_2_; *A*_1_ and *A*_2_ are the transition probabilities of diverse lines; *g*_1_ and *g*_2_ are the statistical weights of the excited states; *E*_1_ and *E*_2_ are the excited state energies of different lines.

From equation (7), it can be found that


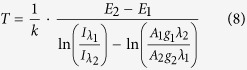


For the two lines whose wave lengths are *λ*_1_ and *λ*_2_, *A*_1_, *A*_2_, *g*_1_, *g*_2_, *E*_1_, *E*_2_ and *k* are the foregone constants, therefore the temperature *T* of the lightning channel could be acquired by equation (8) only by measuring the two spectral line intensities *I*_*λ*_1__ and *I*_*λ*_2__.

Equation (8) provides the direct relation between the lightning intensity and temperature in the lightning channel which can be used as the basic theory of temperature measurement by the atomic emission spectroscopy and as the foundation of the technology by measuring the temperature by relative intensity spectrum of lightning channel.

## The experimental model and the analyses of the data

### The setup of the experimental model

The spectral lines were chosen as the observed objects whose wave length is 648.2 nm in the range of the visible spectroscopy and wave length is 930 nm in the range of the infrared spectroscopy. The optical radiation of lightning is stronger in the visible regime while the continuous radiation is a little weaker in the infrared spectroscopy, as the molecular scattering is weaker than that in the visible band. Thus the infrared spectroscopy band is the best choice to research the optically thin lightning channel. In addition, all the particles in the above two wavelength spectral lines are NII. The parameters, the excitation energy of spectral line and the transition probability, are demonstrated as the Table 1 which satisfy the optically thin lightning channel^18^.

**Figure 1:**
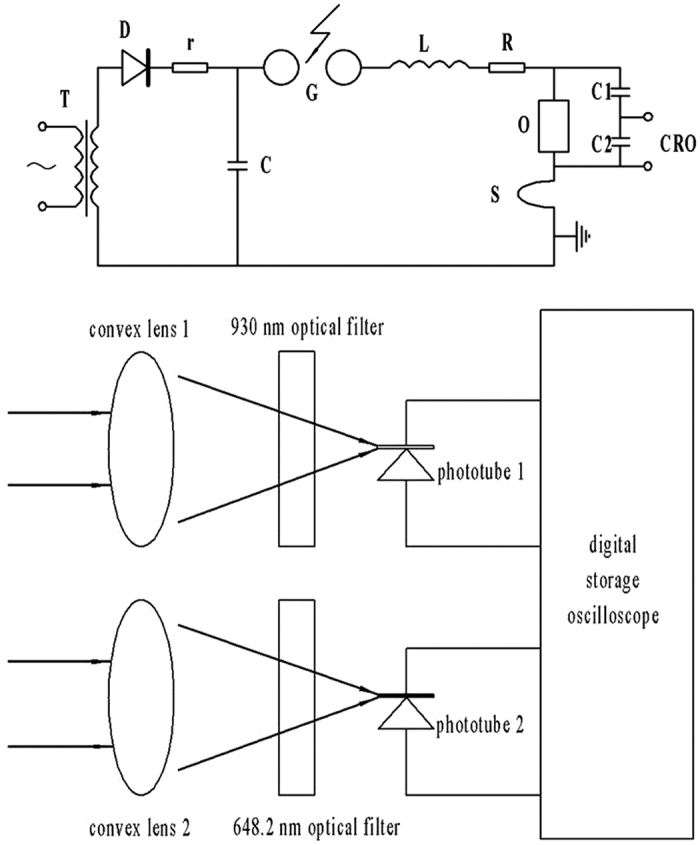
(**a**) The schematic of the ICGS. (**b**) The schematic of spectrum receiving circuit.

The impulse current generator system (ICGS) was used to simulate the lightning current, as shown in Fig. 1(a). The lightning channel forms between the sphere gap *G*, while *C* is the total capacitance of serial capacitors in parallel and *L* is the total inductance of the capacitor, the loop connecting line, the diverter, the rectifier, the sphere gap and the spark on the test product, so does the total resistance *R. G* is the sphere gap, *D* is a silicon stack, *r* is a protective resistance, *T* is a charging transformer, *O* is the test product, *S* is the diverter, *C*_*1*_ and *C*_*2*_ are the capacitances of the diverter, and *CRO* is an oscilloscope. The current value of the lightning channel in the sphere gap is adjusted by the charge voltage value of the capacitance *C*. It is statistic that the distribution of lightning current amplitude has the characteristic of stack, meaning that most lightning current amplitudes are between 10 kA–50 kA, however the amplitude of about 5% of the total cases whose amplitude is more than 96 kA^19^. Therefore, based on the simulating lightning discharged experiments, the temperature of the channel could be diagnosed, and the relationship between the high lightning current and the temperature of the channel can be estimated.

The lightning spectrum receiver is positioned in a distance of 1 m from the sphere gap, as depicted in Fig.1(b). The lightning channel is formed when the gap is broke down, and the lightning rays at 930 nm and 648.2 nm are focused through convex lens 1 and 2, respectively, and are filtered by optical filters. Then the rays irradiate to the phototube 1 and 2, respectively. The phototubes is connected to the oscilloscope, Tektronix TDS 2022B, to transfer the optical signal to the electric signal and select spectrum voltage graphs, while it is used to gain lightning channels with diverse current values that changing the impulse currents of the ICGS. Finally, simulating and analyzing the spectrum voltage graphs, the specific value of the light intensity and the spectral line intensity are obtained whose wavelengths are 930 nm and 648.2 nm.

### The analysis of the data

**Figure 2:**
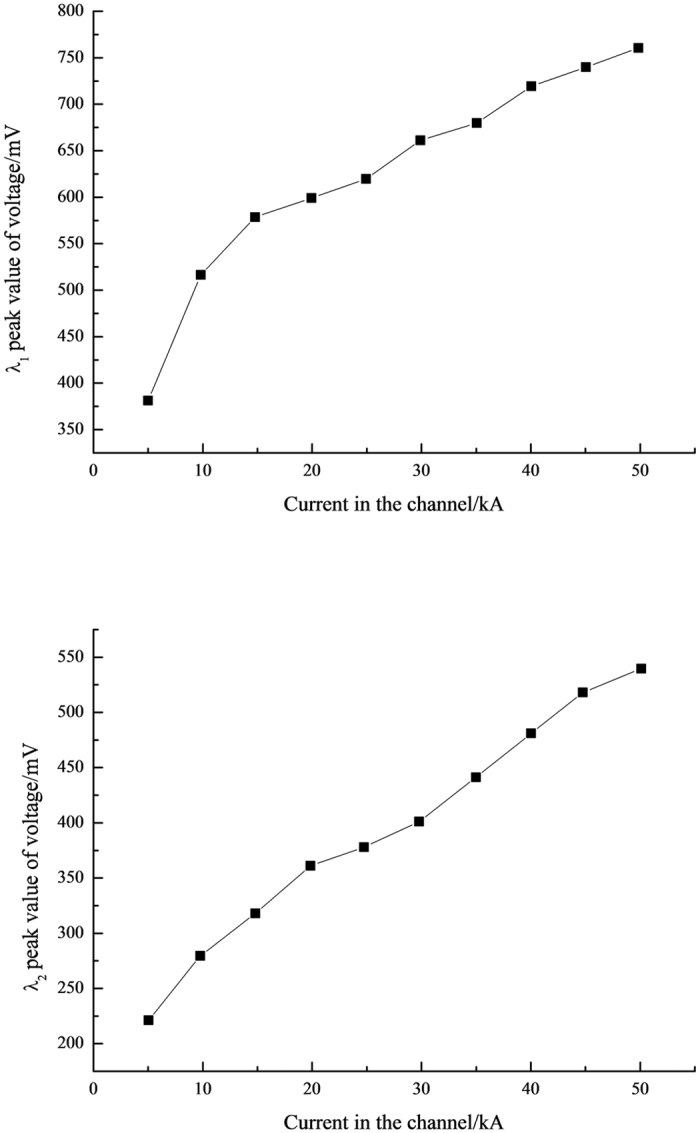
(**a**) The wavelength is 930 nm. (**b**) The wavelength is 650 nm.

**Figure 3:**
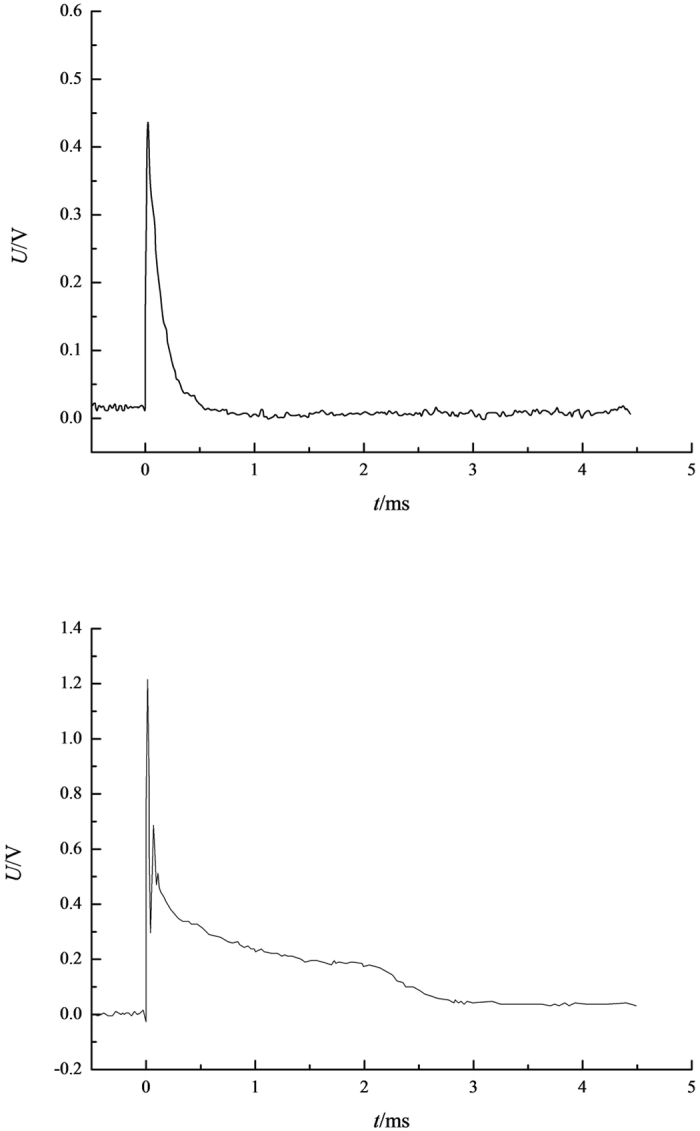
(**a**) The lightning channel current is 5 kA. (**b**) The lightning channel current is 50 kA.

**Figure 4:**
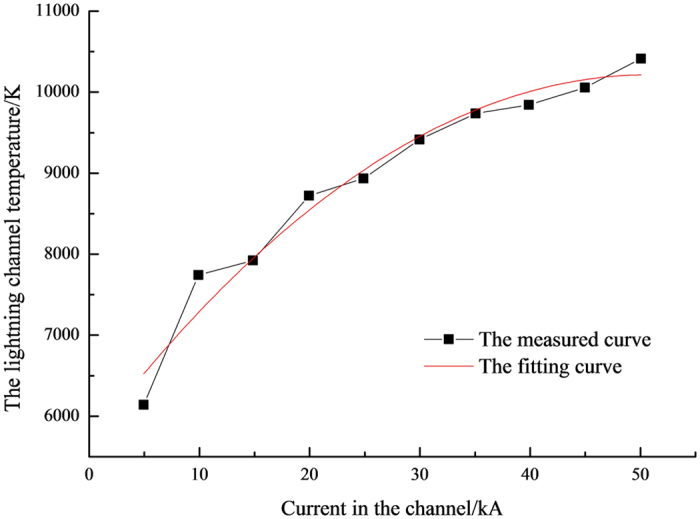
Relationship between lightning channel temperature and current.

According to the spectral lines parameters^7^, the temperature of the lightning channel could be gained by equation (8), exhibited as Table 2. The second and the third column express the peak values of light intensities of two spectral lines with different channel currents, respectively, and the fourth column presents the logarithm fetch on the ratio between two spectral line intensities *I*_*λ*_1__ and *I*_*λ*_2__. From Table 2, the peak values of the light intensities of the two selected spectral lines range from 380 to 760 mV, and 220 to 540 mV, respectively, while the range of temperature is from 6140.8 to 10424 K. The relational graph of spectral line light intensity and the lightning channel current is illustrated in Fig. 2. The light intensity in the infrared band is higher than that in the visible band and it increases with the augment of the lightning channel current values. The light intensities increase gradually in the infrared and invisible band, meaning a positive correlation between the spectral line light intensity and the lightning channel current. The vertical axis expresses the corresponding luminous intensity values by the voltage value. The light intensity and the voltage graph of the spectrum lines in the infrared band have been demonstrated in Fig. 3. Fig. 3(a) is a typical waveform when the current is 5 kA, so does the Fig 3(b) while the current is 50 kA. Not only the peak value but also the energy of the light intensity increase with the augment of the current of the lightning channel as shown in the Fig. 3, which indicates that the area of the voltage waveform aggrandizes. Similar phenomenon emerge in the visible band. Fig. 4 plotted a curve of the current and temperature versus the lightning channel. The current and the temperature are positive correlation, which can be well fitted by a logarithmic formulate.


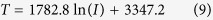


where: *I* is the lightning channel current, kA, whose correlation index *R*_2_ is 98.72%. The correlation index of the fitted curve approaches to 1 closely, thus it is reasonable.

Orville selected two sets spectral lines whose wavelengths are 777.4 nm, 794.7 nm and 844.7 nm, 794.7 nm respectively, obtaining that the temperature is 13000~17000 K of the lightning channel^17^. The infrared spectroscopy, 760 nm~970 nm, was studied by Yuan Ping, during the lightning progress in the Shandong Province, China, while the temperature was 15500~17200 K, whether the spectral line particle was NI or OI^9^. The lightning channel temperature, reckoned by the relative intensity of the neutral atom spectral lines, hardly relied on the primary condition. When the temperature was 15000~18000 K, the radiation intensity would reach the maximum, which was researched by Plooster^20^. The ICGS is used to simulate the lightning discharge channel for temperature measurement, where the temperature yields 6140.8 K–10424 K while the current of lightning channel is 5–50 kA. The relationship between the current and the temperature is presented in equation (9) by fitting, substituting natural lightning channel current values into equation (9), and the calculated temperature is about 14000 K, which show good agreement with the above research results^21^,^22^,^23^,^24^,^25^,^26^,^27^,^28^,^29^.

## Conclusion

In this paper, an approach of temperature measurement for lightning channel is proposed and demonstrated in laboratory. Some conclusion can be drawn as follows.

The peak value of the light intensity in the infrared spectroscopy (930 nm) is higher than that in the visible spectroscopy (648.2 nm) and both of them increase with the current intensity of the lightning channel which means that the peak values of the light intensity of the chosen spectra increase with the augment of the currents.

The temperature varies from 6140.8 K–10424 K when the current changes from 5 kA–10 kA in the lightning channel and there is a good logarithmic relationship between the temperature and the current, *T* = 1782.8ln*I* + 3347.2, the correlation index being 98.72%.

It has good agreement with the relation between the current and the temperature in the natural lightning channel that the relationship between the current and the temperature in the lightning channel which was calculated by the research to the infrared spectroscopy (930 nm) and the visible spectroscopy (648.2 nm), which offers a reference to the lightning physical analysis and the photoelectricity characters.

## Additional Information

**How to cite this article**: Li, X. *et al*. Measuring Method for Lightning Channel Temperature. *Sci. Rep.* **6**, 33906; doi: 10.1038/srep33906 (2016).

## Figures and Tables

**Table 1:** Features of spectral lines with different wavelengths.

Wavelength /nm	Particles in the spectral line	Excited energy E/eV	Statistical weight /g	Transition probability/s^−1^
930	NII	21.8	3	13.2 × 10^8^
648.2	NII	20.4	1	1.21 × 10^8^

**Table 2:** Experimental data of simulated lightning channel.

The lightning channel current /kA	The peak value of*λ*_1_/mV	The peak value of*λ*_2_/mV	In(*I*_*λ*1_/*I*_*λ*2_)	The lightning channel temperature T/K
5	380	220	0.507	6140.8
10	520	280	1.054	7760.7
15	580	320	1.101	7938.7
20	600	360	1.282	8718.4
25	620	380	1.330	8950.4
30	660	400	1.426	9453.1
35	680	440	1.479	9755.5
40	720	480	1.497	9861.9
45	740	520	1.533	10088.8
50	760	540	1.585	10424.0
